# Pterional Craniotomy With Anterior Clinoidectomy for the Resection of a Sphenoid Ridge Meningioma: A Case Report and Two-Dimensional Operative Video

**DOI:** 10.7759/cureus.49379

**Published:** 2023-11-25

**Authors:** Jiahai Ding, Buqing Liang, Liyi Deng, Er Nie, Yang Lu, Jason H Huang, Yong Liu, Lei Wang

**Affiliations:** 1 Department of Neurosurgery, Affiliated Hospital of Xuzhou Medical University, Xuzhou, CHN; 2 Neurosurgery, Baylor Scott & White Health, Temple, USA; 3 Neurosurgery, Baylor Scott & White Medical Center, Temple, USA

**Keywords:** total resection, anterior clinoidectomy, sphenoid ridge, pterional, meningioma

## Abstract

The pterional craniotomy with anterior clinoidectomy is a surgical technique used to resect sphenoid ridge meningiomas. It involves drilling the bone of the anterior clinoid process to gain access to the skull base, including the cavernous sinus and petrous apex particularly. This approach offers several advantages, including excellent exposure of the surgical site, minimal brain retraction, and the ability to visualize and protect critical neurovascular structures. We present a case of a 59-year-old woman presented with headache, dizziness, blurry vision, and unsteady gait for several months. The brain magnetic resonance imaging with gadolinium contrast showed a large space-occupying homogeneously-enhancing lesion at the left skull base, displacing the surrounding structures, including the frontal lobe, temporal lobe, and brainstem. Herein, we present the intraoperative video on a case in which the pterional craniotomy with anterior clinoidectomy that can allow the exposure and resection of the tumor extending into the posterior fossa was utilized for the resection of a large left sphenoid ridge meningioma with brain stem compression.

## Introduction

Meningiomas are generally accepted to arise from arachnoid cap cells within the leptomeninges, accounting for approximately 30% of primary intracranial tumors [1-3]. To date, according to the criteria included in the World Health Organization (WHO) histological classification [4], meningiomas are classified into grades I, II, and III, with higher recurrence and shorter survival times occurring in the higher grade [3]. The Simpson scale is the most significant factor for predicting recurrence risk for resection [5].

Skull base meningiomas involve many complicated and crucial structures [6]. Meningiomas of the sphenoid ridge, the frequent skull base meningiomas, classified into outer (pterional), middle (alar), and inner (deep, clinoidal) lesions, represent higher morbidity, mortality, and recurrence rates [7]. In particular, medial sphenoid ridge meningiomas present a great challenge as they often surround the optic nerve, anterior circulating arteries, cavernous sinus, etc., and compress the brainstem [8].

We present the case with pterional craniotomy with anterior clinoidectomy for the gross total resection of a large low-grade sphenoid ridge meningioma without postoperative adjuvant therapy. This presentation reviewed the case presentation, imaging, surgical anatomy, technique, and postoperative results.

## Case presentation

**Figure 1:**
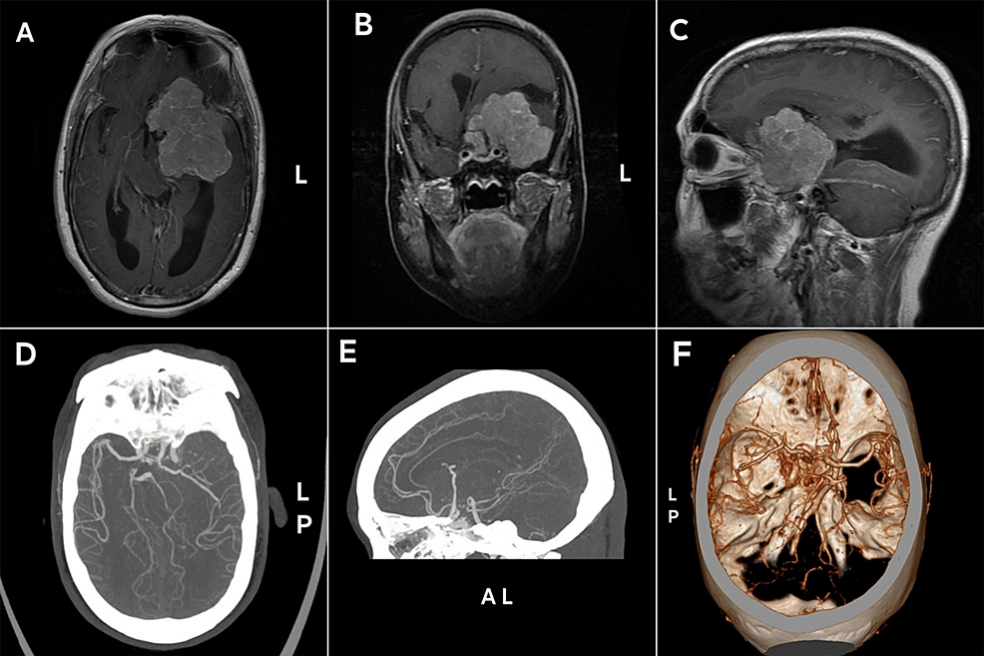
Preoperative images A: contrast-enhanced T1-weighted axial view; B: contrast-enhanced T1-weighted coronal view; C: contrast-enhanced T1-weighted sagittal view; D, E, F: computed tomography angiography coronal and sagittal views, and 3-dimensional reconstruction

A 59-year-old woman presented with headache, dizziness, blurry vision, and unsteady gait for several months was sent to an outer hospital. Magnetic resonance imaging (Figures 1A-C) of the brain showed probable left temporal lobe meningioma, so she was transferred to the hospital. Brain MRI with gadolinium contrast showed a large space-occupying homogeneously-enhancing lesion measuring approximately 7.3x4.9x5.7 cm at the left skull base, displacing the surrounding structures, including the frontal lobe, temporal lobe, and brainstem. In order to further clarify and identify the blood vessels, computed tomography angiography (CTA) of the brain clearly depicted the rightward and upward displacement of the left internal carotid artery terminus, anterior cerebral arteries, and middle cerebral arteries (Figure 1D-F).

Considering the significant mass effect of the tumor, surgical resection was strongly recommended, which allows immediate decompression of the normal brain tissues. The patient underwent a gross total resection of the lesion with a standard left-sided frontotemporal craniotomy: the skin flap and the temporalis muscle were taken as one piece and flipped anteriorly and inferiorly. During the operation, the middle meningeal artery was coagulated. The drilling of the sphenoid ridge and anterior clinoid process provided the neurosurgeon with a wide surgical field. We grinded the sphenoid ridge to the level of the skull base and ensured the integrity of the sphenoid sinus when grinding the anterior clinoid process. After dissecting the Sylvian fissure, the tumor was immediately exposed, and its capsule was devascularized. Subsequently, using a combination of bipolar, microscissors, ultrasonic aspirator, and tumor forceps, the tumor was resected in a piecemeal fashion until the superior orbital fissure was exposed and unroofed to expose the oculomotor nerve. So, the tumor between the oculomotor nerve (CN III) and internal carotid arteries was isolated and resected. Finally, the base of the tumor was removed to decompress the brain stem (see Video 1).

**Video 1:** Surgical procedure ICA - internal carotid artery; N - nerve

**Figure 2:**
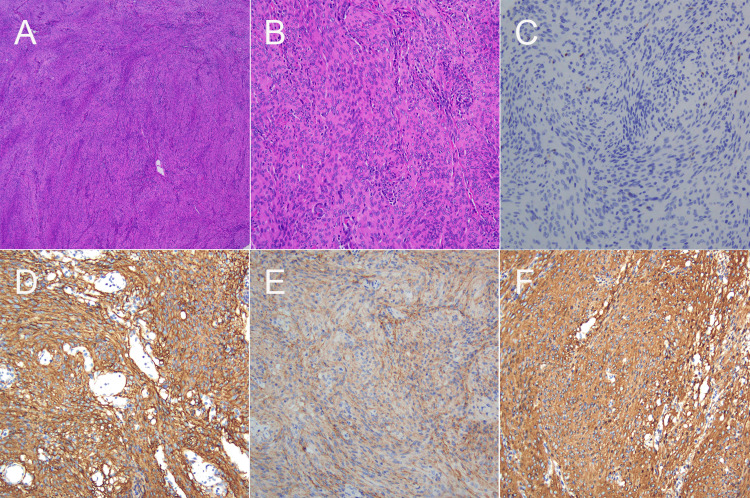
Pathology Hematoxylin and eosin stain (A: x40) and (B: x200); Ki-67 labeling index was 1% (C). The tumor cells were immunohistochemically positive for somatostatin receptor (SSTR) 2 (D: x200),  epithelial membrane antigen (EMA, E: x200) and vimentin (F: x200).

**Figure 3:**
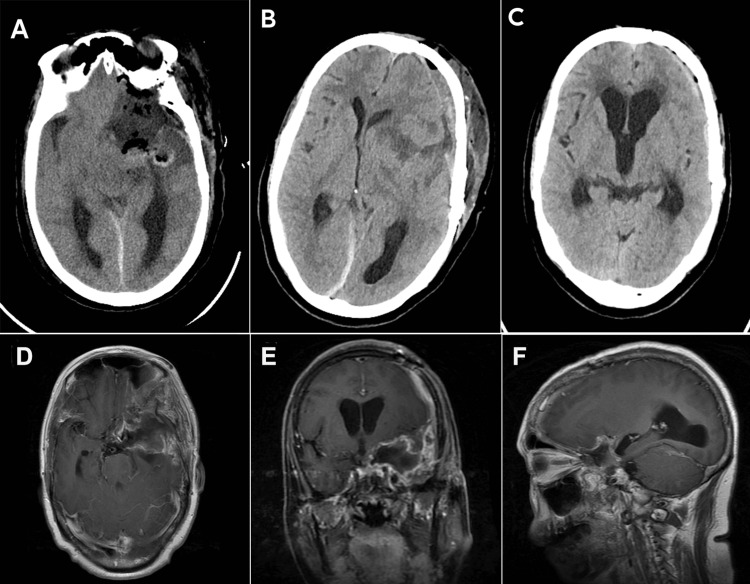
Postoperative images A: postoperative CT at three days; B: postoperative CT at 10 days; C: postoperative CT at one month; D-F: postoperative enhanced MRI at one month

Postoperatively, Simpson grade II resection of the tumor was achieved, and the tumor was pathologically diagnosed as WHO grade I meningioma (Figures 2A-F). Postoperative CT (Figures 3A-C) and MRI (Figures 3D-F) showed the expected performance and gross total resection of the tumor. The midline shift was improved, and the brain stem was decompressed. The patient recovered well without new neurological deficits and was discharged home.

## Discussion

Alternatives to surgical resection for meningiomas include observation, radiation, chemotherapy, and stereotactic needle biopsy [9]. However, neither would be ideal due to the large tumor volume and brain stem compression. In our case, considering the significant mass effect of the tumor, surgical resection was strongly recommended, which allows immediate decompression of the normal brain tissues.

Anterior clinoidectomy allows adequate exposure of the proximal internal carotid artery, optic nerve, sella turcica, and central skull base. Epidural clinoidectomy can protect intradural structures during bone removal, but subdural blood vessels and nerves cannot be directly observed. For medial sphenoid bridge meningiomas, this technique could provide mid- to early-stage control of the tumor base and decompression of the optic nerve. Meanwhile, intradural anterior clinoidectomy is performed under direct vision of the intradural structure, which could avoid unnecessary anterior clinoid process removal and increase the protection of the sphenoid sinus and oculomotor nerve. For tumors with severe bone hyperplasia or ossification variations in the clinoid process ligament, intradural or combined extradural and extradural approaches would be more reasonable [10,11].

Radical resection provides patients suffering from meningiomas with the best prognosis and long-term disease control [12,13]. Sphenoid ridge meningiomas are characterized by deep location, complex structures, and narrow surgical space, leading to a high risk of damaging nerves, blood vessels, and brainstem as well as possibly causing postoperative paralysis, so that completely removing it could be challenging [8,14]. When the tumor is hard and adherent to normal structures, leaving a piece of it intentionally can preserve the patient's postoperative neurological functions [15]. The pterional approach with anterior clinoidectomy for this patient was chosen as it provides great convenience not for the lesion removal in the carotid-oculomotor triangle (COT) but the treatment of the tumor base. The benefits of the surgery include tumor resection and identification of pathology, recovery of neurological functions, and brain stem decompression, whereas, potential risks include cranial nerve injuries and paralysis, major artery injuries and brain infarcts, epilepsy, cerebrospinal fluid (CSF) leak, and infection [8,16].

At present, the Simpson grade scale still holds substantial prognostic value for meningiomas [17], while a grading scale that relies on postoperative MRI imaging that assesses gross total resection (GTR) versus subtotal excision (STR) is being applied [18]. A postoperative MRI of our patient showed expected presentations and gross total resection of the tumor, and she hasn't relapsed until now. Postoperative radiotherapy is not recommended for grade I meningiomas without a high risk of recurrence [19], but based on the high risk of recurrence and mortality after total resection of giant sphenoid ridge meningiomas, whether postoperative adjuvant therapy brings greater benefits deserves further investigation [20]. For our patient, although the lesion was large, gross total resection has been performed, and she recovered well, so close follow-up is required, and radiotherapy or chemotherapy is not necessary for the time being. However, for subtotal resection of grade I meningiomas that are difficult to perform salvage surgery, postoperative adjuvant therapy should be considered.

## Conclusions

In our case, the pterional craniotomy with anterior clinoidectomy approach provided excellent exposure and extent of resection, with important structures such as the oculomotor nerve and internal carotid artery being pretty protected. Meanwhile, we found that combined drilling of the anterior clinoid process through both internal and external dura mater can probably achieve total resection for giant skull base meningiomas, especially those that invade cavernous sinus and petrous apex.
